# Pseudomyxoma peritonei extending to the lower extremity: a case report

**DOI:** 10.1186/s12957-015-0639-x

**Published:** 2015-07-19

**Authors:** Min Wook Joo, Yang-Guk Chung, Soo Young Hur, Ahwon Lee, Chan Kwon Jung, Won-Hee Jee, Jong Ho Kim

**Affiliations:** Department of Orthopaedic Surgery, College of Medicine, St. Vincent’s Hospital, The Catholic University of Korea, Jungbu-daero 93, Paldal-gu, Suwon-si, Gyeonggi-do 442-723 Republic of Korea; Department of Orthopaedic Surgery, College of Medicine, Seoul St. Mary’s Hospital, The Catholic University of Korea, Banpo-daero 222, Seocho-gu, Seoul 137-701 Republic of Korea; Department of Obstetrics and Gynecology, College of Medicine, Seoul St. Mary’s Hospital, The Catholic University of Korea, Banpo-daero 222, Seocho-gu, Seoul 137-701 Republic of Korea; Department of Hospital Pathology, College of Medicine, Seoul St. Mary’s Hospital, The Catholic University of Korea, Banpo-daero 222, Seocho-gu, Seoul 137-701 Republic of Korea; Department of Radiology, College of Medicine, Seoul St. Mary’s Hospital, The Catholic University of Korea, Banpo-daero 222, Seocho-gu, Seoul 137-701 Republic of Korea

**Keywords:** Pseudomyxoma peritonei, Lower extremity, Diagnosis, Therapeutics, Prognosis

## Abstract

Pseudomyxoma peritonei is characterized by mucinous ascites originating from a mucin-producing neoplasm; however, even the definition is still under debate. Tumor deposits extend and ultimately engulf the entire cavity, causing death from cachexia due to limited intestinal movement. Here, we report a unique case of an 80-year-old woman with pseudomyxoma peritonei, which extended to the lower extremity mimicking infectious condition. The patient survived for a long time without bowel obstruction despite having the histologic subtype that has an unfavorable prognosis. The extremity lesion was treated with limited extensive surgery. The origin of the disease and the mechanism of extension to the extremity could not be clarified. Clinicians should be aware of the original disease entity and this unusual presentation and determine its mechanism and the best management strategy.

## Background

Pseudomyxoma peritonei (PMP) is a clinical syndrome characterized by mucinous ascites that result from rupture of a mucin-producing neoplasm [1–3]. However, the origin, pathology, treatment, prognosis, and even the very definition are still under debate [2]. While PMP is currently thought to be associated mainly with neoplasm of appendix [3–9], several convincing cases have demonstrated other primary organs of origin such as the ovary, urachus nest, and colon [10, 11]. In 1995, three distinct categories of PMP were proposed on the basis of pathological findings [10]. Recently, the paramount importance of histopathological subtype in determining the outcome was confirmed, and peritoneal mucinous carcinoma (PMCA) subtype was reported to be an independent predictor of poorer overall survival based on a large case series with long-term follow-up [12]. PMP usually manifests as tumor deposits throughout the peritoneum [13]; it then engulfs the entire peritoneal cavity and limits intestinal movement. Patients ultimately die of cachexia if untreated [3, 14, 15].

To the best of our knowledge, no case of PMP extension to the lower extremity has ever been reported. Therefore, the presentation, mechanism, and complications are unknown, and the management strategy, including the appropriate surgical margin and adjuvant treatment for the extremity lesion, are undetermined. In this paper, we share our experience with this rare presentation.

## Case presentation

An 80-year-old woman presented to our institution with odorous discharge from an opening in her swollen right thigh. In the previous 4 years, her right thigh had swollen up. Three months prior to her visit, an opening formed spontaneously at the medial aspect of the thigh, and jelly-like material began to ooze steadily. Two months before her visit, an odorous discharge began to exude from the opening. Physical examination revealed fluctuation in the right lower quadrant of the abdomen, the medial aspect of the right thigh, and the posterior aspect of the right calf.

Fourteen years previously, the patient had undergone an operation for an intra-abdominal mass at another institution. The operation record mentioned a bilateral salpingo-oophorectomy with massive adhesiolysis and excision of a large 20 × 19 × 18-cm mass in the retroperitoneal space. There was no mention of the appendix in the record, and the patient had not undergone appendectomy before the operation. The pathological record reported a mucinous borderline left ovarian tumor with extensive pseudomyxoma ovarii and severe tubo-ovarian adhesion. Although the patient had noted progressive abdominal expansion with dull pain in the right lower quadrant 8 years previously, diagnostic studies such as computed tomography (CT) and ultrasonography (US) [16] were not performed.

**Fig 1:**
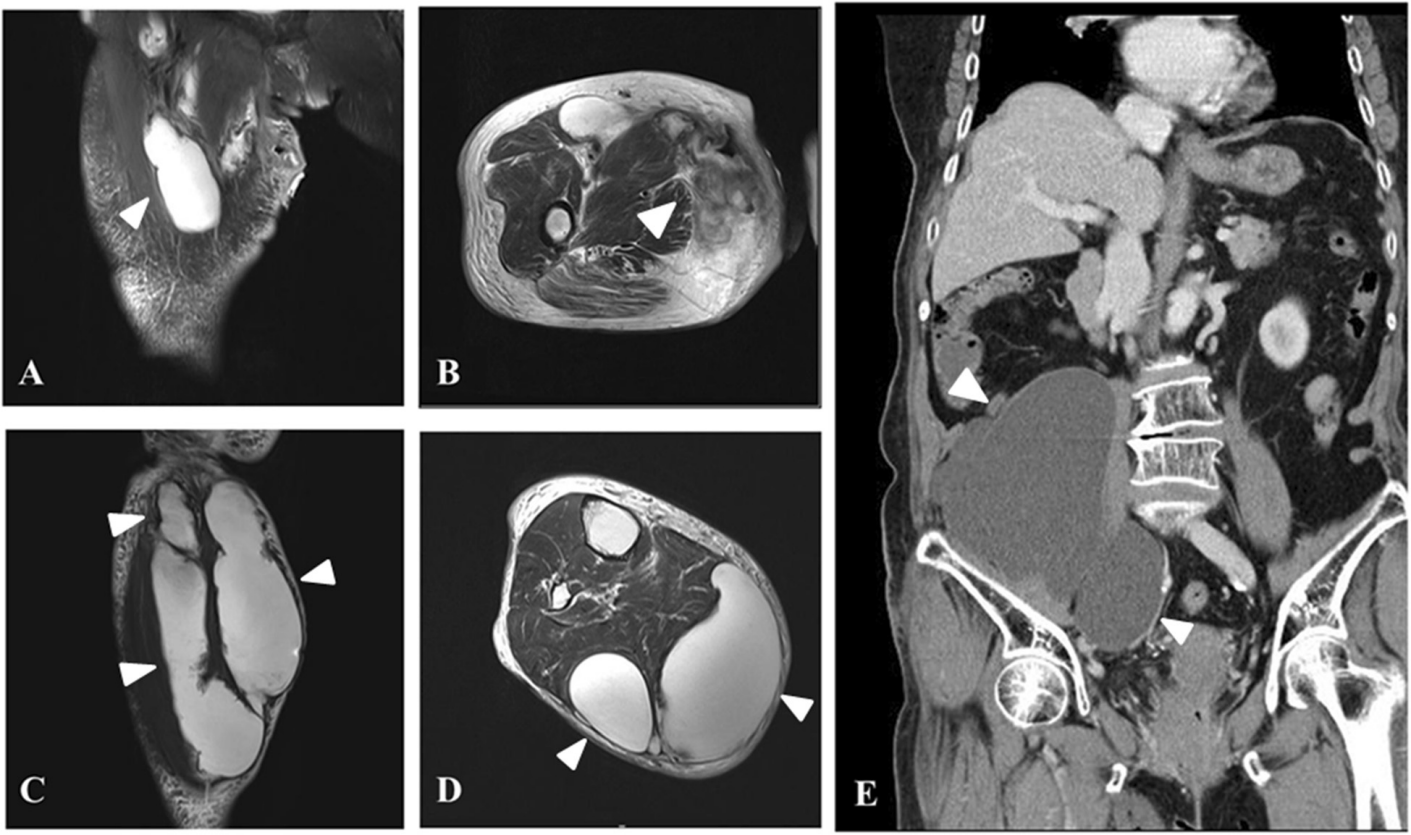
Image findings. **a** A T2-weighted coronal view magnetic resonance image (MRI) of the right thigh shows a cystic lesion mainly involving the anterior compartment, especially the sartorius muscle (an *arrowhead*). **b** A T2-weighted axial view MRI reveals lesions mainly involving the medial compartment along the fascia of the hamstring muscles (an *arrowhead*). **c** A T2-weighted coronal view of MRI of the right calf shows multiloculated and lobulated lesions mainly involving the posterior compartment (*arrowheads*). **d** A T2-weighted axial view MRI reveals a lesion along the superficial fascia (*arrowheads*). **e** A coronal view abdominal computed tomography scan shows a large multiloculated cystic mass occupying the right retroperitoneal space (*arrowheads*)

A magnetic resonance image of the right thigh showed a fluid-containing lesion in contact with the surface and an opening in the medial compartment. In addition, there was a multiloculated, lobulated lesion mainly involving the anterior and medial compartment of the thigh and extending distally along the fascia of the hamstring muscles (Fig 1a, b). A magnetic resonance image of the calf also revealed a similar lesion chiefly involving the posterior compartment along the deep fascia (Fig 1c, d). An abdominal CT scan showed a large multiloculated cystic mass occupying the right retroperitoneal space and extending to the right thigh (Fig 1e). There were no definite abnormal findings on the chest CT scan. Together, these imaging studies revealed extension of the lesion from the abdomen to the calf. The patient’s initial blood tests revealed abnormal results including a white blood cell (WBC) count of 11,820/mm^3^, 87.5 % segmented neutrophil, an erythrocyte sediment rate of 96 mm/h, 5.89 mg/dL C-reactive protein, 178 mg/dL fasting blood sugar, 4.5 g/dL total protein, 2.4 g/dL albumin, and 50 U/L aspartate transaminase. The urine analysis and sediment examination noted abnormal results including leukocytosis, a positive occult blood, and WBC and red blood cell count of 30 to 49 and 20 to 29 per high-power field.

**Fig 2:**
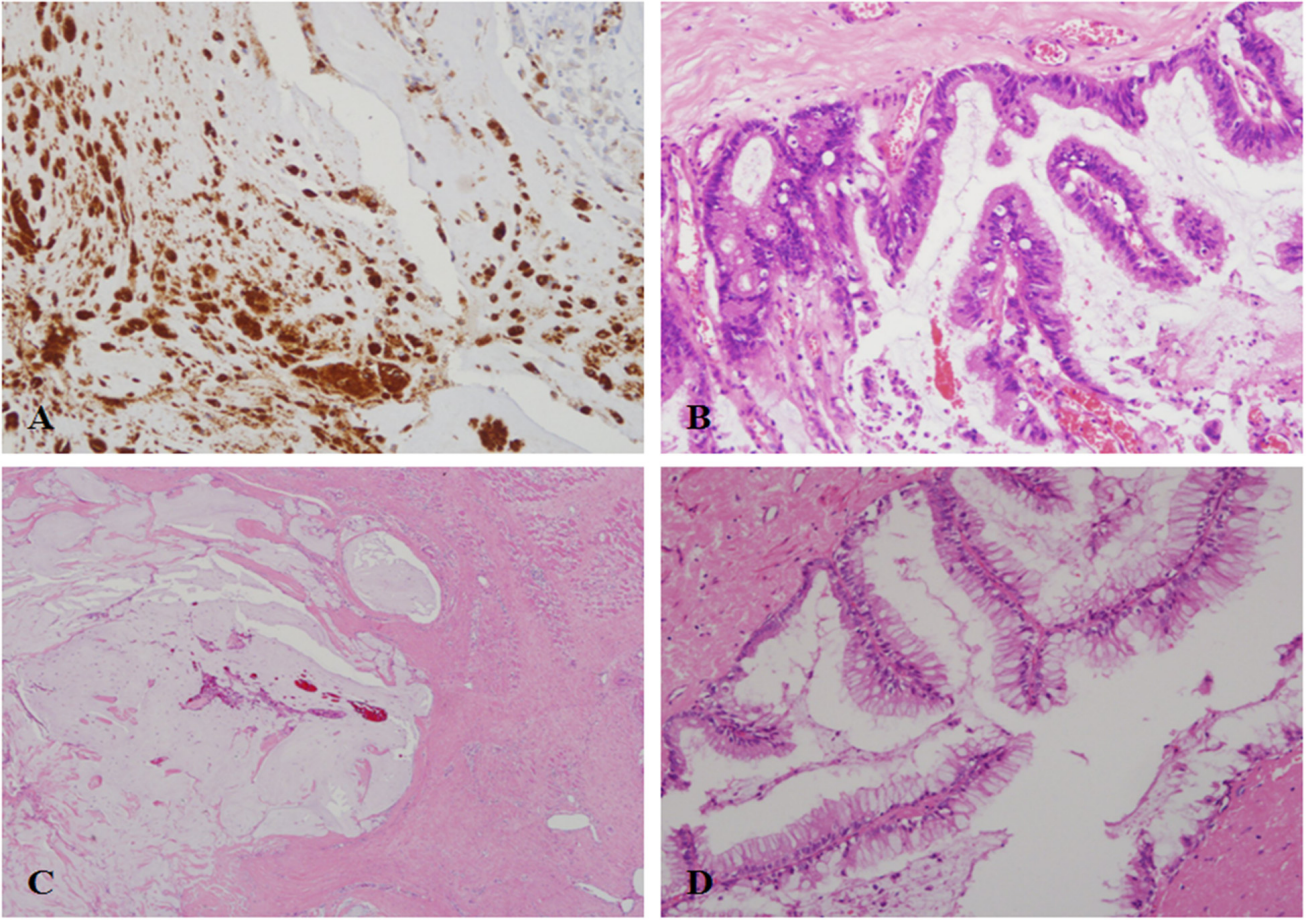
Pathologic findings. **a** Mucinous material obtained from the right thigh shows a carcinoembryonic antigen-positive immunoreaction (carcinoembryonic antigen antibody stain, original magnification ×200). **b** A pathology slide demonstrates low-grade pseudomyxoma peritonei. The malignant epithelium appears bland, and tumor cells look deceptively bland with papillary tufting (hematoxylin and eosin stain, original magnification ×200). **c** Dissecting mucin without tumor cells was observed in the soft tissue of the right thigh (hematoxylin and eosin stain, original magnification ×40). **d** A previous pathology slide demonstrates pseudomyxoma ovarii. The malignant epithelium appears bland, and tumor cells look deceptively bland with papillary tufting

Immediately after admission, we started intravenous antibiotic administration, which included 2.25 g of piperacillin and tazobactam four times a day and 1 g vancomycin once every 96 h. Two weeks after admission, a suspected infected portion of the thigh lesion was excised, and tracks connecting from the thigh to the peritoneum were ligated. After the operation, administration of 2.25 g of piperacillin and tazobactam four times a day was continued. Staphylococcus epidermidis grew when mucinous material obtained intra-operatively from the lesion was cultured. Pathological examination of the excised specimen demonstrated acellular mucinous materials with a carcinoembryonic antigen-positive immunoreaction (Fig 2a).

Two weeks after the first surgery, follow-up blood test results demonstrated improvement of the infectious condition in the thigh. The WBC count, segmented neutrophil, erythrocyte sediment rate, C-reactive protein, fasting blood sugar, total protein, albumin, and aspartate transaminase were 4640 /mm^3^, 51.8 %, 11 mm/h, 1.09 mg/dL, 112 mg/dL, 5.7 g/dL, 2.9 g/dL, and 24 U/L, respectively. On urine analysis and sediment examination, the WBC and red blood cell count were 1 to 3 and 4 to 9, respectively, per high-power field.

**Fig 3:**
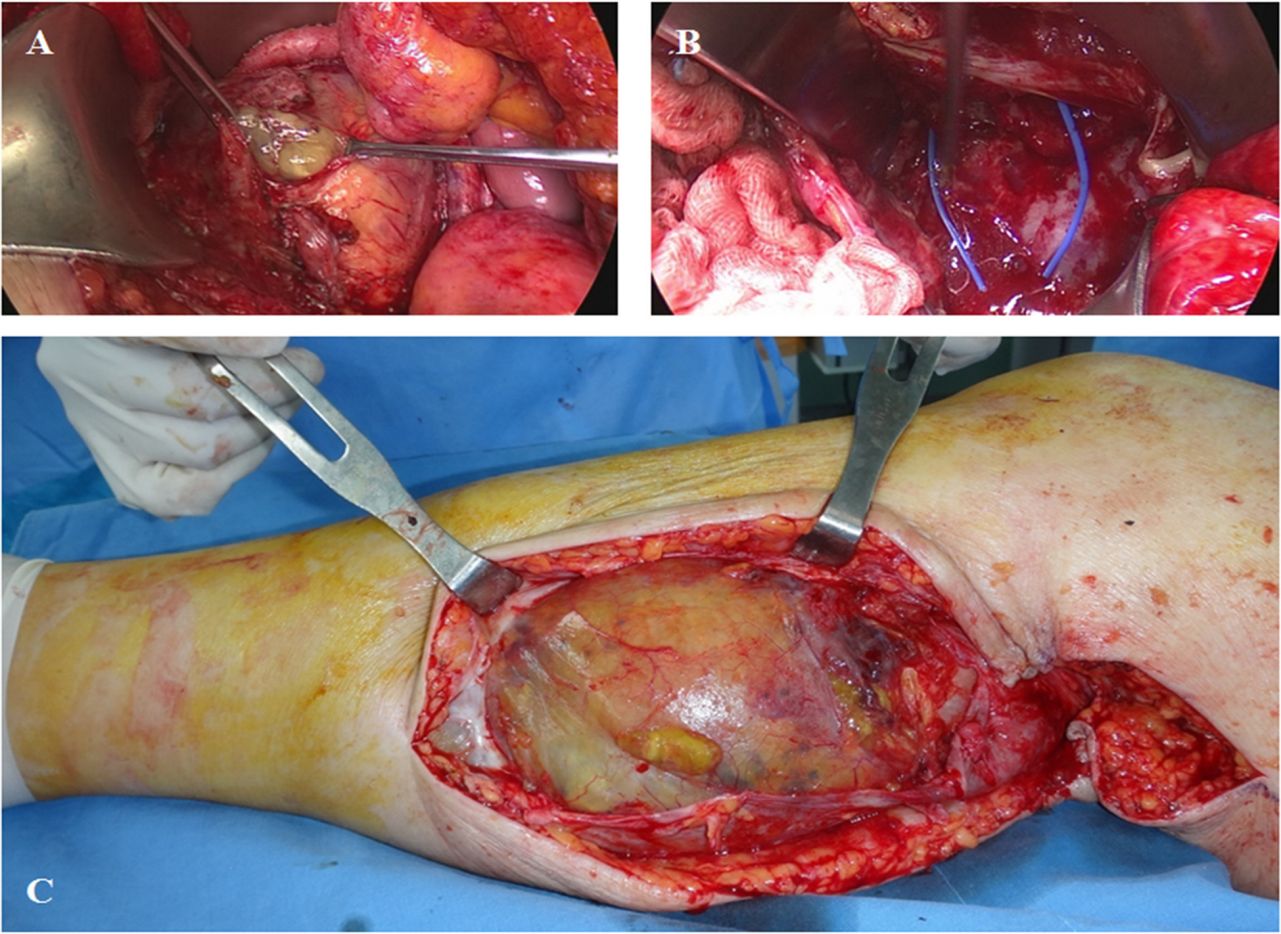
Intra-operative findings. **a** Mucus ascites is seen gushing from the abdominal lesion. **b** Multiple intra-muscular and interfascial connections were observed at the level of the inguinal ligament. **c** A huge cystic lesion with mucus was observed in the calf

A second operation was performed for all the lesions from the abdomen to the calf by a collaborative team of orthopedic surgeons and gynecologists. No bowel obstruction was observed, and the appendix was not seen. A large volume of mucinous material was suctioned from the abdominal lesion (Fig 3a). Multiple intra-muscular and interfascial connections between the abdomen and lower extremity were observed at the level of the inguinal ligament (Fig 3b). Cytoreductive surgery with peritonectomy was performed for the intra-abdominal lesion. The lower extremity lesion was marginally removed; it had a thin capsule and was mostly composed of mucinous material (Fig 3c). Four hours after the operation, the patient underwent cardiopulmonary resuscitation following a hypovolemic shock due to massive intra-operative bleeding. She recovered with proper management without any acute sequelae.

Five days after the second surgery, no bacterial growth was observed in a culture of intra-operatively obtained mucinous material. Pathological examination showed low-grade mucinous carcinoma peritonei with the lining epithelium in the intra-abdominal lesion (Fig 2b) and only acellular mucinous material in the lower extremity lesion (Fig 2c). The patient’s postoperative course was uneventful, and she was discharged 2 weeks after the definitive operation. Thereafter, she sent us the pathology slides from 14 years previously, which were re-examined by pathologists at our institution. They revealed pseudomyxoma ovarii (Fig 2d). The patient did not report any abdominal symptoms or swelling in the lower extremity at the most recent follow-up, which was 2 years after the final operation. She was able to walk with a cane, and the Musculoskeletal Tumor Society functional rating for the lower extremity was 73 %.

### Discussion

The origin of PMP is still controversial. PMP is currently thought to be associated mainly with mucinous epithelial neoplasms of the appendix [3–9]. However, several convincing cases have been published with a different primary organ of origin, such as the ovary, urachus nest, or colon [10, 11]. Because our patient had not undergone an appendectomy before the operation in 1999 and there was no mention on the appendix in the operation record, we could not confirm the appendix as the origin of PMP on histological grounds. Despite the previous pathological report, the ovaries were normal and did not appear to be the origin of the tumor based on the review of the previous slides. We, therefore, assume that the appendix was the organ of origin in this case.

PMP usually manifests with tumor deposits throughout the entire peritoneal cavity. This characteristic pattern of dissemination can be explained by the redistribution phenomenon, which is associated with the intra-peritoneal fluid current and gravity [13]. In the end stage of disease, PMP engulfs the entire peritoneal cavity. Intestinal movement becomes limited because of an excessive amount of mucinous tumor mass, and bowel obstruction becomes imminent. Patients ultimately die of cachexia when untreated [3, 14, 15].

CT and US have been reported to be useful in detecting PMP [3, 17, 18]. While CT remains the gold standard for diagnostic imaging, US is inexpensive, readily available, well tolerated, and can identify most common PMP findings such as ascites and omental caking. Our patient did not have any diagnostic examination despite abdominal symptom, and a relapse was diagnosed barely after development of the lower extremity lesion.

In 1995, Ronnett et al [10] described three distinct categories of PMP, namely, diffuse peritoneal adenomucinosis (DPAM), PMCA, and an intermediate/discordant subtype (PMCA-I/D) on the basis of pathological findings. They reported that 5-year overall survival rates of 75 % in DPAM, 50 % in PMCA-I/D, and 14 % in PMCA when the same surgeon treated PMP tumor patients uniformly. Recently, Chua et al. [12] examined the outcome of nearly 2300 patients from 16 institutions worldwide who were treated uniformly over an 18-year period and confirmed the paramount importance of the histopathological subtype with respect to outcome. Five-year overall survival rates were 81 % for DPAM, 78 % for hybrid tumors, and 59 % for PMCA. Multivariate analysis showed that the PMCA subtype was an independent predictor of poor overall survival.

Although the intra-peritoneal lesion in this case was diagnosed as low-grade PMCA and optimal treatment was not performed for a long time, our patient did not report any symptoms associated with bowel obstruction. Despite a huge lesion, her clinical course was modest over a long period, and death by cachexia was avoided. We assume that the extension of the intra-peritoneal lesion to the lower extremity acted as an expansion of the peritoneal cavity and prevented such an outcome. When PMP is progressing, the characteristic visceral and mesenteric sparing of PMP becomes visible intra-operatively and is related to a favorable prognosis after surgery [19], as in our case.

The proper treatment for PMP remains uncertain; however, the combination of aggressive cytoreductive surgery and hyperthermic intra-peritoneal chemotherapy seems to improve the outcome [20]. Nevertheless, extensive surgical resections to achieve complete tumor eradication are associated with high morbidity and mortality rates and may decrease the quality of life without any survival benefit, particularly in cases of extensive high-grade diseases [21–23]. In contrast, less aggressive surgery may be optimal in cases of extensive low-grade disease, as in the extremity lesion of our patient if the benefit compensates for surgical morbidity [24]. Chemotherapy may cause side effects, particularly surgical complications, as a result of bone marrow toxicity [25]. As in the case of peritoneal lesions, we had to consider balancing treatment benefit and morbidity in the extremity lesion. A wide excision encompassing large areas of muscle and parts of neurovascular structures could have rendered the extremity nonfunctional, especially with adjuvant treatments. In addition, it may have caused more severe hypovolemia. Even if we had chosen more aggressive management with careful support from anesthesiologists, it would have been difficult to achieve R0 margin because the mucinous contents and thin capsule extended from the abdomen to the calf. The first surgery had already contaminated the thigh area. The advanced age of the patient also added complexity; therefore, we performed less extensive surgery without any adjuvant treatment for the extremity lesion.

Although the cause of PMP extension to the lower extremity has an important clinical significance, we could not explain why the extension occurred only in this case. The fluidity of mucinous contents and the gravity might have contributed to the formation of the lower extremity lesions as an extension of tuberculous psoas abscess into the thigh. Further studies to elucidate the mechanism are required.

## Conclusions

We reported a rare case of PMP extension to the lower extremity. Clinicians should understand the original disease entity of PMP, be aware of this unusual presentation and complication, and determine the best management strategy by balancing the benefits against possible morbidity. We also expect further studies to elucidate the mechanism of PMP extension to the lower extremity.

## Consent

Written informed consent was obtained from a legal guardian of the patient for publication of this case report and any accompanying images. A copy of the written consent is available for review by the editor in chief of this journal.

## Abbreviations

CT: computed tomography
DPAM: diffuse peritoneal adenomucinosis
PMCA: peritoneal mucinous carcinoma
PMCA-I/D: Intermediate/discordant subtype
PMP: pseudomyxoma peritonei
US: ultrasonography
WBC: white blood cell
